# A First Insight into the Developability of an Immunoglobulin G3: A Combined Computational and Experimental Approach

**DOI:** 10.1021/acsptsci.4c00271

**Published:** 2024-07-15

**Authors:** Georgina B. Armstrong, Alan Lewis, Vidhi Shah, Paul Taylor, Craig J. Jamieson, Glenn A. Burley, William Lewis, Zahra Rattray

**Affiliations:** † Drug Substance Development, GlaxoSmithKline, Gunnels Wood Road, Stevenage SG1 2NY, U.K.; ‡ Computational and Modelling Sciences, GlaxoSmithKline, Gunnels Wood Road, Stevenage SG1 2NY, U.K.; § Large Molecule Discovery, GlaxoSmithKline, Gunnels Wood Road, Stevenage SG1 2NY, U.K.; ∥ Department of Pure and Applied Chemistry, University of Strathclyde, Glasgow G1 1XL, U.K.; ⊥ Strathclyde Institute of Pharmacy and Biomedical Sciences, University of Strathclyde, Glasgow G4 0RE, U.K.

**Keywords:** antibody, viscosity, developability, IgG1, IgG3, computational models, protein−protein interactions, monoclonal antibody

## Abstract

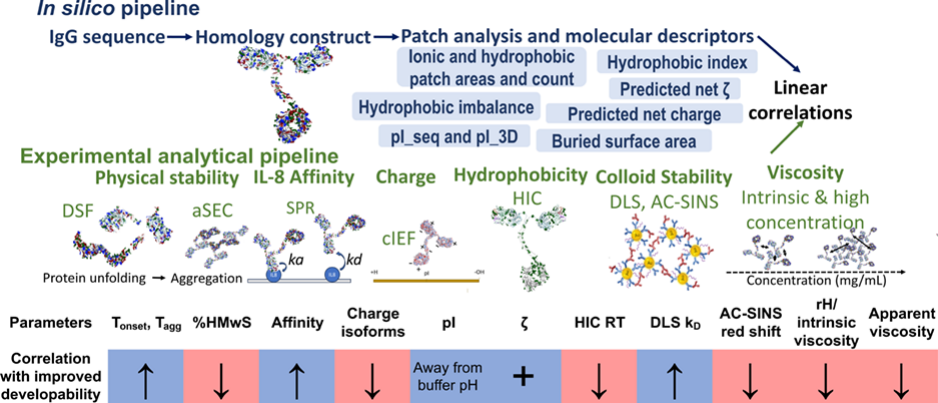

Immunoglobulin G 3 (IgG3) monoclonal antibodies (mAbs) are high-value scaffolds for developing novel therapies. Despite their wide-ranging therapeutic potential, IgG3 physicochemical properties and developability characteristics remain largely under-characterized. Protein–protein interactions elevate solution viscosity in high-concentration formulations, impacting physicochemical stability, manufacturability, and the injectability of mAbs. Therefore, in this manuscript, the key molecular descriptors and biophysical properties of a model anti-IL-8 IgG1 and its IgG3 ortholog are characterized. A computational and experimental framework was applied to measure molecular descriptors impacting their downstream developability. Findings from this approach underpin a detailed understanding of the molecular characteristics of IgG3 mAbs as potential therapeutic entities. This work is the first report examining the manufacturability of IgG3 for high-concentration mAb formulations. While poorer conformational and colloidal stability and elevated solution viscosity were observed for IgG3, future efforts controlling surface potential through sequence-engineering of solvent-accessible patches can be used to improve biophysical parameters that dictate mAb developability.

Antibody-based therapies possessing high specificity and superior efficacy have gained tremendous traction and growth in the biopharmaceuticals sector. Antibodies exert their pharmacological activity via a range of biological mechanisms, including but not limited to, direct blockade or activation of cell signal transduction pathways; Fc-mediated functions (antibody-dependent cell-mediated cytotoxicity,^1^ complement-dependent cytotoxicity, antibody-dependent cell phagocytosis), and immune activation.^2^ The molecular diversity of monoclonal antibody isotypes and their subclasses can be harnessed to achieve different mechanisms of action in combating disease. Immunoglobulin G (IgG), the most abundant antibody isotype, can be further categorized as IgG1, IgG2, IgG3, and IgG4 subclasses in descending order of their prevalence in human serum.^3^

While the sequence homology of IgG subclasses is highly conserved (>90%), each of these subclasses possesses a unique hinge region length, differences in the number of interchain disulfide bonds, and Fc-effector functionality.^1^ The molecular diversity of IgG subclasses and their involvement in mediating responses to different immunologic stimuli reflect the differing functional roles of IgG subclasses, affording their application in targeting a diverse antigen landscape. From a biotherapeutic perspective, there has been growing recognition in recent years that the biomolecular properties of the different IgG subclasses correlate with improved developability characteristics, particularly in the context of targeting otherwise inaccessible biological targets.

Of the four IgG subclasses, IgG3 has the highest binding affinity for FcγRs but is not routinely explored for therapeutic indications due to its historical suboptimal physicochemical stability profile and immunogenicity risk.^2^ However, the IgG3 hinge region influences the flexibility of this subclass of antibody, enabling IgG3 to interact more effectively with target antigens that are expressed at lower abundance.^3^ While both IgG1 and IgG3 play key roles in mediating immune responses, their structural differences lead to variations in their interactions with FcγRs and subsequent immune effector functions.^4^ IgG1 and IgG3 interact differently with most immune receptors (FcγR), triggering various immune effector mechanisms such as phagocytosis or antibody-dependent-cell-mediated cytotoxicity, which can offer therapeutic potential in immuno-oncology applications.^5^

IgG1 and IgG3 differ mostly based on the composition of their hinge region, which alters the extent of their ability to activate the immune system. IgG1 mAbs contain two interchain disulfide bonds in the hinge region, while IgG3 mAbs have 11 interchain disulfides. These structural differences influence their effector functions, with the IgG3 longer hinge length contributing to a combined greater accessibility to antigens and Fcγ receptors, resulting in more potent opsogenic activity.^2^

Beyond differences in their biological properties, each IgG subclass is associated with developability challenges, in the context of resistance to fragmentation, aggregation propensity, and elevated solution viscosity at high concentrations.^6^ Although IgG1 mAbs exhibit superior stability under different pH conditions and in response to mechanical stress, they are more prone to fragmentation. However, IgG2 and IgG4 mAbs by comparison are less prone to fragmentation but are more susceptible to aggregation.^6^

A dearth of IgG3 candidates in biopharmaceutical pipelines has been attributed to a lack of binding to protein A hampering downstream processing efforts,^7,8^ lack of *in vivo* stability resulting from proteolytic susceptibility, short plasma half-life necessitating a higher dosing frequency to achieve therapeutically relevant levels,^9^ and immunogenicity concerns.^2,5^ However, with recent biotechnological advances in antibody sequence-based engineering, formulation strategies, and advancements in downstream processing, these challenges can be mitigated. Mitigating such risks requires the development of IgG3-based molecular descriptors and biophysical properties under mAb formulation conditions, which identify key features that enhance as well as hinder downstream developability.

Here, a comprehensive study is presented to address the current knowledge gap of IgG3 developability characteristics, arising from sequence and structural differences to the IgG1 subclass. In this study, we analyze a computationally derived set of molecular descriptors of an anti-IL-8 IgG1 and IgG3 pair. This mAb pairing possesses identical variable domains. A comprehensive framework is then constructed to align the computational prediction of IgG1 and IgG3 sequences with measured experimental parameters evaluating their self-association behavior and solution viscosity at high formulation concentrations (>100 mg/mL).

## Experimental Section

### Computational Methods

*In silico* homology modeling and antibody molecular descriptor calculations were performed in the Molecular Operating Environment (MOE) software, version 2020.0901 (Chemical Computing Group, Montreal, Canada).

#### Homology Modeling of Anti-IL-8 IgG1 and IgG3

For both IgG1 and IgG3 molecules, full sequences of the heavy and light chains were inputted as the FASTA format into MOE (sequence editor) and annotated with a Kabat numbering scheme, with identical variable chain sequences. Constant chains were selected from the IMGT Repertoire database (https://www.imgt.org/IMGTrepertoire/), with accession numbers J00228 (IGHG1*01) and M12958 (IGHG3*01) for IgG1 and IgG3, respectively. For the IgG1 molecule, the Antibody modeler in MOE (version 2020.0901) was used to search for similar sequences with solved antibody structures to form the templates used for homology constructs. The variable fragment (Fv) of anti-IL-8 is published as PDB ID: 5OB5 (fAb complex with GroBeta). Fv fragments and full IgG structures were modeled by selecting “variable domain” and “immunoglobulin” model types, respectively. The immunoglobulin model type used the 1IGY PDB structure as a template to model the fragment crystallizable (Fc) region. A refinement gradient limit value of 1 was applied, and C-termini were capped with neutral residues and superimposed to confirm structure alignment. For the IgG3, a different approach of independently modeling each antibody component was required due to the absence of resolved IgG3 structures arising from the long hinge length. A new template hinge was generated independently using a mouse IgG2A (pdb: 1IGT) as the second and fifth C–C disulfide bridges were in the same positions as the IgG3 hinge sequence (Supporting Information). This sequence was copied a further three times to generate four modules of the hinge. The Homology modeler in MOE (version 2020.0901) was used to generate 10 refined homology models for the hinge (Supporting Information). Each parameter was normalized to rank the geometric quality per model:
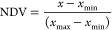
1where NDV is the normalized value for all geometric quality scores, except for the packing score, which was computed using eq 2.
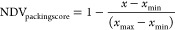
2

The lowest heavy atom root-mean-square deviation to the average position of intermediate models and the lowest normalized score model were selected. A human Fc (pdb: 6D58) was imported for the Fc fragment, and the fragment antigen-binding regions (Fabs) were modeled via the Antibody modeler tool in MOE (version 2020.0901) from the anti-IL-8 IgG3 Fab sequence, with Fab selected as the model type. A 100% match to PDB ID 5OB5 was found as the variable sequence was the same between IgG1 and IgG3, with only a five-residue sequence difference in the constant regions of the Fab. All components were then joined manually, and the join energy was minimized.

#### Patch Analysis of Anti-IL-8 IgG1 and IgG3 Homology Constructs

The protein patch tool in the MOE was applied to each homology construct to identify electrostatic and hydrophobic surface patches. To aid visualization of smaller surface patches, we set the following parameter thresholds: hydrophobic cutoff: ≥ 0.09 kcal/mol, hydrophobic min area: ≥ 30 Å^2^, charge cutoff: ≥ 30 kcal/mol/C, charge min area: ≥ 30 Å^2^, and probe sphere radius: 1.8 Å.

#### Predicted Physicochemical Descriptors

We computed a range of molecular descriptors (Supporting Information) for each full IgG1 and IgG3 model using the MOE *Protein Properties* tool. A NaCl concentration of 0.1 M was selected to represent the formulation buffer ionic strength at pH 6. *Hydrophobic imbalance* and *buried surface area* values were generated through BioMOE (version 2021-11-18, Chemical Computing Group, Montreal, Canada).

### Generation and Biophysical Analysis of Anti-IL-8 IgG1 and IgG3

#### IgG1 and IgG3 Expression and Downstream Purification

Chinese Hamster Ovary (CHO) K1 GS-KO (glutamine-synthetase-knockout) cells were used to express IgG1 and IgG3. Heavy and light chain sequences were codon optimized and inserted into plasmids with CMV promoters by Atum Biosciences (Newark, CA, US). Plasmids were transfected via nucleofection with Leap-in Transposase mRNA into Chinese Hamster Ovary (CHO) cells and maintained under selection conditions (no glutamine supplement) to generate stable pools. A fed-batch production process for 15 days with nutrient/glucose feeds every two or 3 days was deployed to increase the expression of anti-IL-8 IgG1 and IgG3. Cell culture bulk samples were fully clarified and then purified with an initial Protein L capture step followed by cation exchange polishing. Purified IgG1 and IgG3 were then concentrated, diafiltered, and exchanged into a formulation buffer containing histidine, trehalose, and arginine (pH 6) to a final target concentration of ≥150 mg/mL.

### Biophysical Analysis of Anti-IL-8 IgG1 and IgG3

#### Analysis of Identity

Peptide mapping was used to verify the full sequence identity for IgG1 and IgG3 (Supporting Information).

#### Analysis of Purity

Analytical size-exclusion chromatography (aSEC) with UV detection was deployed for the monomeric purity assessment of anti-IL-8 IgG1 and IgG3. A TSKgel Super SW3000, 4.6 mm × 300 mm (TOSOH Bioscience, United States) column was used with Agilent 1260 series HPLC (CA, US). Samples were prepared in water at 5 mg/mL and ran at 0.2 mL/min with a mobile phase containing 400 mM NaCl (pH 6.8). Chromatogram processing and integration were performed in The OpenLab CDS Data Analysis software (version 2.6, Agilent, California, US). The target monomeric purity of ≥95% was met by both anti-IL-8 IgG1 and IgG3 molecules, and aSEC was used to monitor physicochemical stability by monitoring changes in the chromatogram.

#### Hydrophobic Interaction Chromatography (HIC) of IgG1 and IgG3

The hydrophobicity of IgG1 and IgG3 was assessed using HIC on an Agilent 1260 series HPLC instrument (Agilent, California, US), coupled with UV detection (214 and 280 nm). A PolyLC PolyPROPUL 4.6 × 100 mm column was used, and to achieve separation based on net hydrophobicity, stepwise gradients of mobile phase B (low salt, with 50 mM ammonium sulfate) followed equilibration with mobile phase A (high salt, 1.3 M ammonium sulfate). IgG1 and IgG3 samples were analyzed at 1 mg/mL (5 μL injection volume) and a 0.7 mL/min flow rate.

#### Capillary Isoelectric Focusing (cIEF)

Charge distribution profiles of anti-IL-8 IgG1 and IgG3 were assessed *via* capillary isoelectric focusing using an iCE3 instrument (Protein Simple, US). A range of pI markers (pI 3.85–8.77, Bio-Teche, Protein Simple, USA) were used to capture all acidic and basic isoforms for both molecules. To help prevent aggregation, 2 M urea was added to the 1:1 ampholyte mixture (pH 3–10 and pH 8–10.5). The method entailed a prefocus voltage of 1,500 V; an autosampler/transfer capillary temperature of 15 °C; a 10–12 min focus voltage of 3,000 V; UV detection at 280 nm; a sample injection pressure of 2,000 mbar; a prefocus time of 1 min; and a focus time of 10–12 min. The Empower 3 software (v4, Waters, US) was used for data analysis of peaks.

#### Electrophoretic Light Scattering

A Malvern Zetasizer (Malvern Panalytical, Malvern, UK) with a 633 nm laser was used to measure the zeta potential of the IgG1 and IgG3 pairs by electrophoretic light scattering. Default settings included a 120 s equilibration time, automated attenuation, and 10–100 measurement runs. There was a 60 s pause between each measurement, and three technical replicate measurements were performed.

#### Determination of IgG1 and IgG3 Self-Interaction

The self-association propensity of anti-IL-8 IgG1 and IgG3 was measured by Affinity-Capture Self-Interaction Nanoparticle Spectroscopy (AC-SINS). Goat antihuman Fc and whole goat antibodies (Jackson ImmunoResearch, PA, USA) were prepared in 20 mM acetate buffer (pH 4.3), then mixed and incubated with 20 nm gold particles (Ted Pella Inc., CA, USA, concentration 7.0 × 10^11^ particles/mL). Test samples were prepared at 50 μg/mL in phosphate-buffered saline (PBS) and 99 μL was added to 11 μL of nanoparticles in a 96-well plate, resulting in a final solution concentration of 50 μg/mL test mAb, 10x bead: anti-Fc conjugate and 0.02 mg/mL PEG2000. Plates were agitated, incubated for 2.5 h, and gently centrifuged to remove air bubbles. Absorbance measurements were read using a Pherastar FSX (BMG Labtech Ltd., Germany) plate reader, and spectra were analyzed with MARS software (v3.32, BMG Labtech Ltd., Germany). Differences in plasmon wavelengths for each sample were calculated from smoothed best-fit curves. Experimental cutoffs included a < 535 nm wavelength for negative controls (i.e., buffer).

#### Diffusion Self-Interaction Parameter

A Stunner (Unchained Laboratories, CA, USA) dynamic light scattering setup was used to measure analyte hydrodynamic size, polydispersity, and diffusion coefficient. Data were analyzed using the Lunatic & Stunner Client software (version 8.1.0.254). The measurement temperature was set as 25 °C with five, 10-s measurements acquired with a corresponding 1% extinction coefficient of 1.55 AU*L/(g*cm) for all samples. Custom dispersant settings were applied (viscosity 1.26 cP and refractive index 1.33 at 20 °C) and both molecules were prepared in formulation buffer (0.5–20 mg/mL). The Lunatic & Stunner software (v8.1.0.244) was used for data export, and corresponding diffusion coefficients were used to calculate interaction parameters (*k*_D_) using linear regression plots.
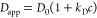
3where *D*_app_ refers to the apparent diffusion coefficient, *D*_0_ is the self-diffusion coefficient at infinite dilution, and *k*_D_ is the interaction parameter.

Exponential fits for diffusion coefficients and logarithmic fits for hydrodynamic radius over the test concentration range were used to calculate theoretical viscosities, adapted from the generalized Stokes–Einstein equation:
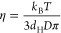
4where η is the theoretical dynamic viscosity (cP), *k*_B_*T* is the Boltzmann constant at 298 K, *d*_H_ is the *Z*-ave diameter, and *D* is the diffusion coefficient.

#### Zeta Potential of Anti-IL-8 IgG1 and IgG3

A Malvern Zetasizer (Malvern Panalytical, Malvern, UK) with a 633 nm laser was used to measure the zeta potential of the IgG1 and IgG3 pair by electrophoretic light scattering. Each sample (refractive index 1.59) was prepared to 5 mg/mL in formulation buffer (pH 6.0, refractive index 1.33, viscosity at 1.26 cP) and a method was set up with equilibration time of 120 s, automatic attenuation, and up to 100 runs per sample. A 60 s pause was also set between sample runs (a minimum of three technical replicates was performed).

#### Analysis of Unfolding Temperatures

Differential scanning fluorimetry was performed on IgG1 and IgG3 anti-IL-8 molecules using a Prometheus NT.48 setup (NanoTemper Technologies, Germany) with back-reflection technology. The intrinsic fluorescence from unfolding events exposing tyrosine and tryptophan residues was monitored via the 350/330 nm intensity ratio.^10^ A temperature ramp of 2 °C/min from 20 to 95 °C was performed. Both samples were assessed at concentrations ∼150 mg/mL and unfolding temperatures of antibody domains (*T*_m1_, *T*_m2_, and *T*_m3_) detected from first-derivative peaks of the 350/330 nm fluorescence intensity ratio. The first derivative peak of the scattering profile marked the aggregation temperature (*T*_agg_) values.

#### Measurement of Solution Viscosity

Viscosity curves were obtained using a VROC Initium instrument (Rheosense, United States). The measurement protocol was optimized using the “Auto” shear rate function, with fixed shear rates in the 100–2000 s^–1^ at each test concentration. Data were filtered to only include transient curves with steady plateaus with no drift and pressure over sensor position linear fits of *R*^2^ ≥ 0.998. Various models were used to fit the viscosity data. First, the exponential-growth equation was applied:
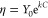
5where η is the dynamic viscosity (cP), *Y*_0_ is the intercept, *k* is the rate constant, and *c* is the concentration of antibody (mg/mL).

Another model, developed by Tomar et al.^11,12^ was deployed to fit the viscosity data:
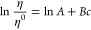
6where η is the dynamic viscosity (cP), η^0^ the buffer viscosity (cP) set at 1.13, *c* is the concentration, and ln *A* is the intercept of the slope *B*, when ln(η/η^0^) is plotted against concentration.

Finally, a modified Ross–Minton model was used to fit the viscosity-concentration profiles:
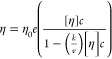
7where η is the dynamic viscosity (cP), η_0_ is the buffer viscosity (cP) set at 1.13, *c* is the concentration (mg/mL), [η] is the intrinsic viscosity, *k* is the crowding factor, and *v* the Simha shape parameter. The [η], *k*, and *v* parameters were estimated using the generalized reduced gradient (GRG) nonlinear solver function to determine the local optimum reducing the sum of squared errors.

For intrinsic viscosity [η] measurements, multiple priming segments were set up followed by 10 replicates at the maximum shear rate of 23,080 s^–1^. Formulation buffer and anti-IL-8 formulations in the 5–50 mg/mL concentration range were measured to determine the relative viscosities (η_rel_) from which the specific (η_sp_) and reduced viscosities (η_red_) could be calculated (Supporting Information). The intrinsic viscosity was calculated from the linear regression of η_red_ over the sample concentration range tested, from which the Huggins coefficient was derived (eq 7).
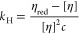
8where *k*_H_ is the Huggins coefficient, η_red_ is the reduced viscosity (cP) which is η_sp_/*c*, [η] is the intrinsic viscosity (cP), and *c* is the sample concentration (mg/mL).

The uncertainty of *k*_H_ was calculated from the propagation of the error equation:
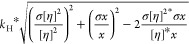
9where [η]^2^ is the squared intrinsic viscosity, σ[η]^2^ is the error of squared intrinsic viscosity, *x* is the slope determined from the linear regression of η_red_ versus concentration, and σ*x* is the error of the slope.

#### Statistical Analysis

JMP Pro (v16.0.0, 2021) was used for multivariate analysis of computational predictions and measurement data to determine correlations between molecular descriptors and experimental parameters. We used GraphPad Prism (v5.04) for constructing graphs and performing unpaired *t*-test statistical analysis.

## Results

### Patch Analysis of Homology Constructs of Anti-IL-8 IgG1 and IgG3

**Figure 1:**
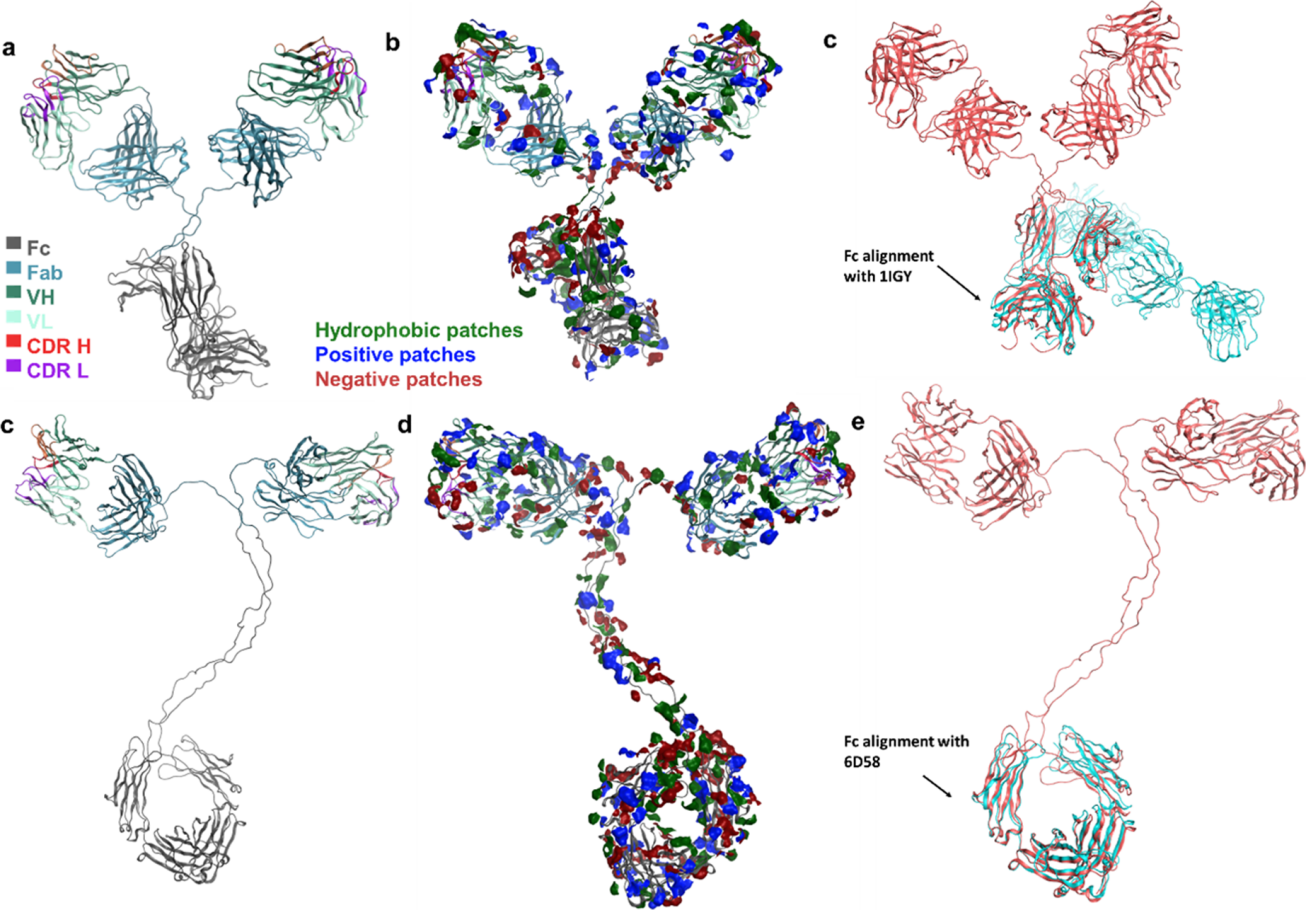
Homology constructs of the full IgG1 and IgG3 molecules. (a) Full IgG1 structure, (c) full IgG3 structure, (b, d) patch analysis of IgG1 and IgG3 homology constructs, and (c, e) Fc templates for IgG1 and IgG3.

Solvent-accessible charge and hydrophobicity distribution profiles mAb self-association propensity that can promote aggregation.^13−15^ Disruption of hydrophobic patches has been previously correlated with reduced viscosity,^16,17^ driven by reduced native and non-native aggregation events.^18^ Furthermore, charge asymmetry between heavy and light chains has been correlated to increased self-association propensity, with increased electrostatic interactions.^13,19,20^ Therefore, we sought to assess the hydrophobic and electrostatic surface patch distribution profiles for the anti-IL-8 IgG1 and IgG3 pair using full IgG homology constructs (Figure 1). Since the variable regions for both antibodies were similar, any differences occurring in the surface potential distributions were attributed to differences in the constant region between the molecules. Overall, both antibodies possessed a high proportion of hydrophobic patches (42 and 37%, respectively), with distinct differences in electrostatic patch (i.e., positive, and negative patch) distributions deriving predominantly from the increased residue exposure of the larger Fc domain of IgG3 (Supporting Information). The lowest energy conformation or the 62-residue IgG3 hinge region homology model was chosen (Supporting Information), contributing to 11 and 9% of the overall negative patch and positive residue contributions, respectively, in comparison to the 4 and 1% contributions from the IgG1 hinge (Supporting Information).

### Biophysical Analyses of anti-IL-8 IgG1 and IgG3

#### Confirmation of Identity and Purity of Anti-IL-8 IgG1 and IgG3

To compare the biophysical properties of IgG3 with those of IgG1, a combined comprehensive pipeline consisting of computationally predicted molecular descriptors and experimental biophysical analyses was used. We analyzed the correlations between *in silico* and experimental charges, including hydrophobicity and colloidal parameters, and viscosity predictions and measurements. Both IgG1 and IgG3 sequence identities were confirmed with LC–MS peptide mapping (Supporting Information).

#### Antigen Binding Affinity of Anti-IL-8 IgG1 and IgG3

The antigen affinity for the anti-IL-8 IgG1 and IgG3 antibody pair was assessed *via* surface plasmon resonance (SPR) (Table 1). Both molecules showed affinity (*K*_D_) for the IL-8 antigen with comparable association (*k*_a_) and dissociation (*k*_d_) rates (within the same order of magnitude). This demonstrated that the sequence and structural differences of the IgG3 constant domain compared to those of IgG1 had little influence on the Fv affinity for the target antigen.

**Table 1:** Antigen (IL-8) Binding Kinetics for IgG1 and IgG3 Assessed *via* Surface Plasmon Resonance (SPR)^a^

molecule	1:1 binding kinetics				kinetics (Χ^2^)
	*k*_a_ × 10^5^ (M^–1^ s^–1^)	*k*_d_ × 10^–4^ (s^–1^)	*K*_D_ (nM)	*R*_max_ (RU)	Χ^2^
IgG1	3.84 (±0.12)	10.27 (±0.98)	2.67 (±0.16)	15.57 (±0.38)	1.57 (±0.62)
IgG3	2.41 (±0.18)	9.17 (±0.05)	3.82 (±0.26)	14.63 (±0.15)	1.69 (±0.27)

a Corresponding (mean ± standard deviation) binding on-rate (*k*_a_), binding off-rate (*k*_d_), the equilibrium dissociation constant (KD), the maximum response (*R*_max_), and goodness of fit (Chi-squared) of the 1:1 binding model (*N* = 3).

#### Short-Term Physical Stability Profiles of Anti-IL-8 IgG1 and IgG3

**Figure 2:**
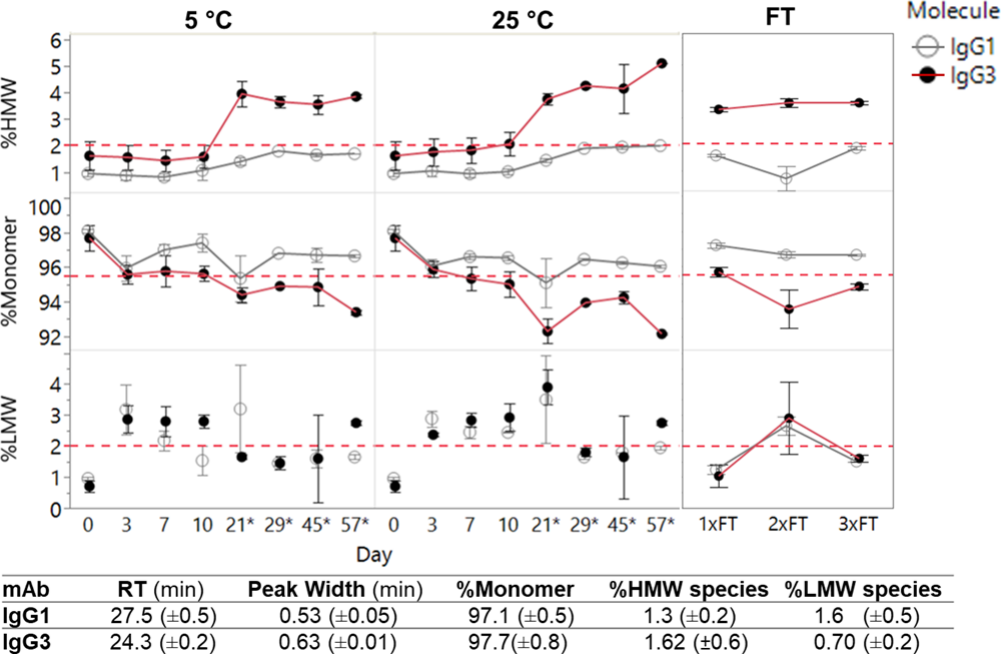
Reduced stability after freeze–thaw cycling and at 25 °C over 57 days for IgG3 compared to IgG1. Analytical size exclusion chromatography (aSEC) was used to monitor the monomeric purity of mAb 1 IgG1 and IgG3 over 57days at 5 and 25 °C. Freeze–thaw stability was also assessed through three cycles. *aSEC data from day 21 to 57 was after one freeze–thaw cycle. Red dotted lines represent thresholds flagging changes in physical stability of mAbs. Corresponding monomeric purity and aggregate content as analyzed by aSEC on day 0 for both molecules (bottom). Error bars represent standard deviations per sample, *N* = 2. Abbreviations: HMwS: high molecular weight species, LMwS: low molecular weight species, FT: freeze–thaw.

To be therapeutically viable, mAb formulations must have a solution phase stability of up to two years at refrigerated temperature and several hours under ambient storage conditions. A short-term stability study (up to 57 days) was conducted to assess relative changes in anti-IL-8 IgG monomeric purity from day 0 under refrigerated and ambient storage conditions and through three freeze–thaw cycles (Figure 2). IgG3 showed a significant reduction in monomer purity from day 0 (surpassing the 2% high molecular weight species threshold) when held at 25 °C by day 7, which could be attributed to an increased level of soluble aggregate formation. This increased aggregation was exacerbated after freeze–thaw cycling, particularly when held at 25 °C.

**Figure 3:**
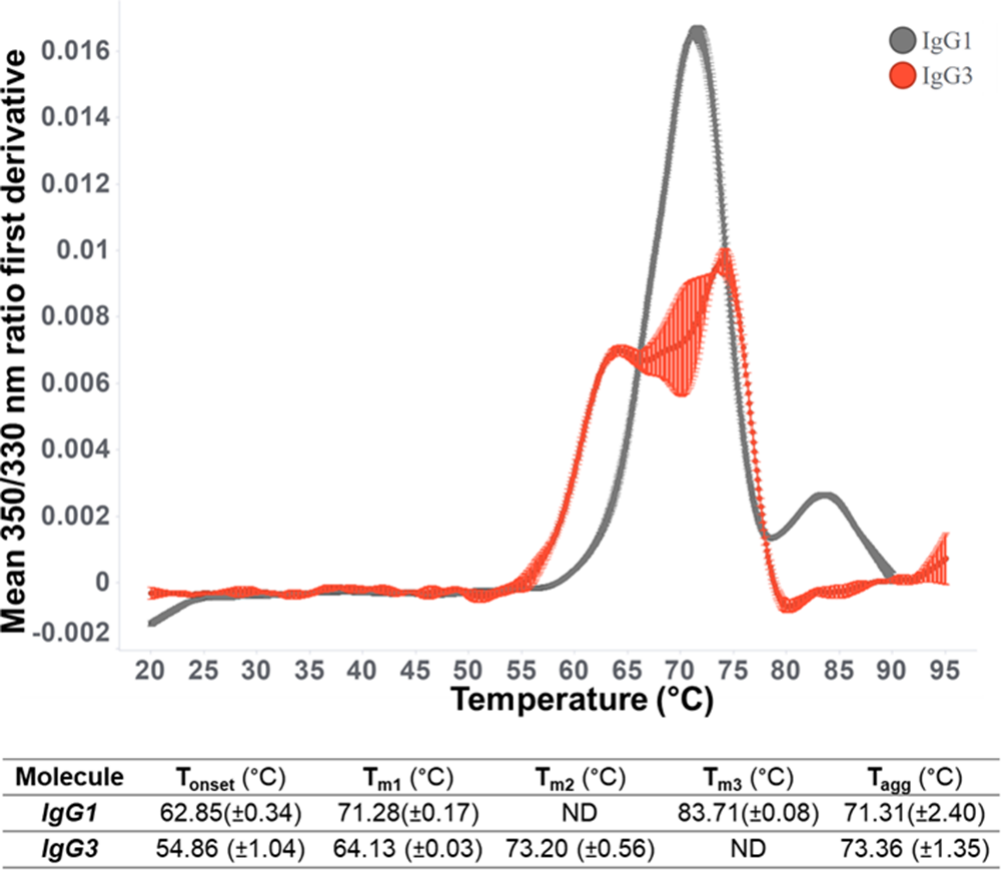
IgG3 shows reduced conformational stability compared to IgG1 at high concentrations. Thermal unfolding profiles were determined for anti-IL-8 IgG1 and IgG3. Mean first derivatives from the 350/330 nm ratio over a 20–95 °C temperature ramp reported as the mean (±standard deviation). *N* = 3.

Differential scanning fluorimetry (DSF) has been used as a surrogate for the assessment of mAb conformational stability and resistance to aggregation in previous work.^200,201^ Here, intrinsic fluorescence DSF was used to compare the unfolding temperatures of IgG1 and IgG3 (Figure 3), with a lower temperature for the unfolding onset (*T*_onset_) and first unfolding event (*T*_m1_) being detected for IgG3, as well as significant changes in the IgG3 thermal profile. No significant differences were detected for the temperature of aggregation onset (*T*_agg_), with IgG3 showing distinctly different scattering intensity profiles compared to IgG1 (Supporting Information).

#### Anti-IL-8 IgG3 Has a Positive Charge under Formulation Conditions

**Figure 4:**
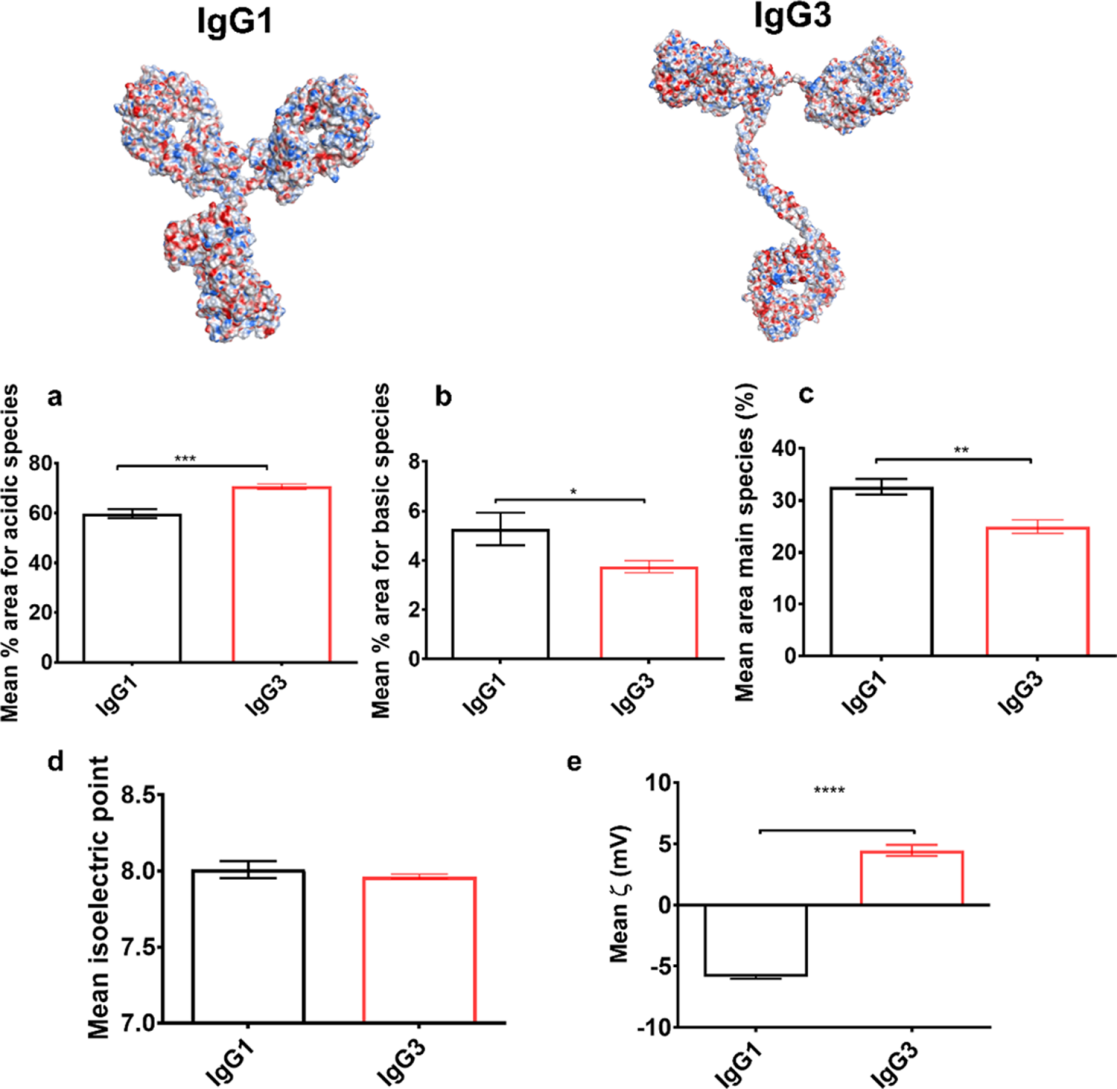
Different surface potential profiles were obtained for anti-IL-8 IgG1 and IgG3 predictions, which yielded comparable measured isoelectric points. Poisson–Boltzmann surface electrostatics were mapped onto homology constructs of anti-IL-8 IgG1 and IgG3, indicating regions of negative and positive charge density. Charge heterogeneity assessed *via* capillary isoelectric focusing (cIEF), (a) acidic isoforms (b) basic isoforms, (c) main species, (d) mean isoelectric point (pI), and (e) zeta potential. Unpaired *t* test **** denotes a *P* < 0.0001, *** *P* < 0.001, ** *P* < 0.01, * *P* < 0.1). Error bars represent standard deviation. Effect sizes were large (Cohen’s *d* > ± 0.8 standard deviations) for mean % acidic species (*d* = 3.5), mean % basic species (*d* = −1.5), mean % main species (*d* = −2.5), and mean ζ (*d* = 15.4).

**Figure 5:**
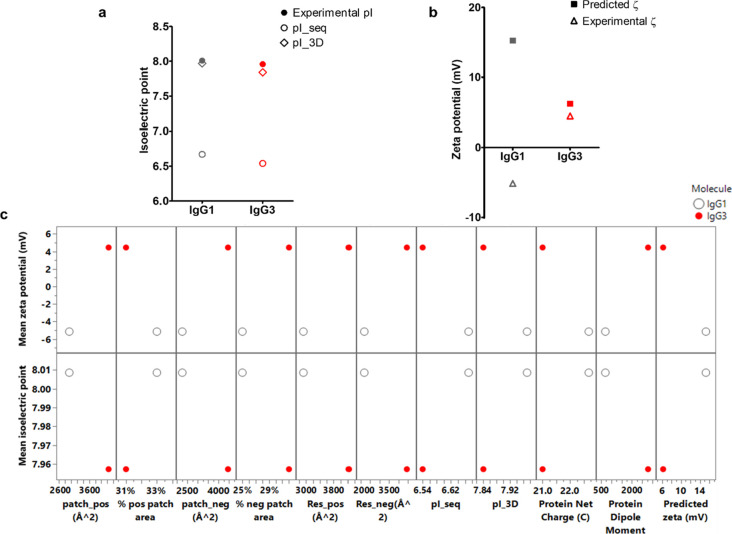
Correlating charge differences to *in silico* charge descriptors for anti-IL-8 IgG1 (gray) and IgG3 (red). (a) The theoretical sequence-based pI was significantly lower than the experimentally measured pI. (b) Zeta potential was measured at 5 mg/mL, demonstrating significant differences in electrical potential at the slipping plane between IgG1 (net negative charge) and IgG3 (net positive charge). Predicted zeta potential (computed at pH 6.0, and 0.1 M NaCl) showed poor correlation with measured zeta potential values. (c) Pair-wise comparisons between charge based in silico descriptors and experimental pI and zeta potential values was performed. Increased positive patch (patch_pos) and negative patch (patch_neg) areas, increased residue contributions to ionic patches (res_pos and res_neg), and decreased net charge aligned with experimental charge values.

Solvent-accessible electrostatic patch distribution profiles of mAbs have previously been linked to changes in protein–protein interactions as a driver of self-association behavior and elevated solution viscosity at high mAb formulation concentrations.^21,22^ We investigated how the predicted differences in electrostatic patch distribution profiles translated to measured charge parameters for anti-IL-8 IgG1 and IgG3 molecules (Figures 4 and 5). Comparable isoelectric points (pIs) (Figure 4d) were measured for IgG1 and IgG3; however, charge heterogeneity differences were observed with an increased proportion of acidic isoforms for IgG3 (Figure 4a), accompanied by an increased proportion of predicted negatively charged patches in the constant domain. IgG1 and IgG3 showed significant differences in the mean measured zeta potential in formulation buffer at pH 6.0, with IgG3 having a positive zeta potential, whereas IgG1 had a negative zeta potential (Figure 4e).

The sequence and structure-based theoretical pIs predicted for IgG3 were slightly lower than those for IgG1, but the structure-based pI (pI_3D) directly correlated with experimental pI (Figure 5a). The positive measured zeta potential (ζ) for IgG3 aligned better to predicted ζ, compared to the negative ζ for IgG1, which was predicted to be positive (Figure 5b). This suggests discrepancies between the effective charge of the molecules in the pH 6 formulation buffer and the net charge that separated the main species from the capillary isoelectric point. The slight reduction observed in the measured isoelectric point and increased measured ζ for IgG3 correlated with increased ionic patch area descriptors and reduced net charge (Figure 5c).

#### Anti-IL-8 IgG3 Exhibits a Lower Degree of Hydrophobicity Compared to IgG1

**Figure 6:**
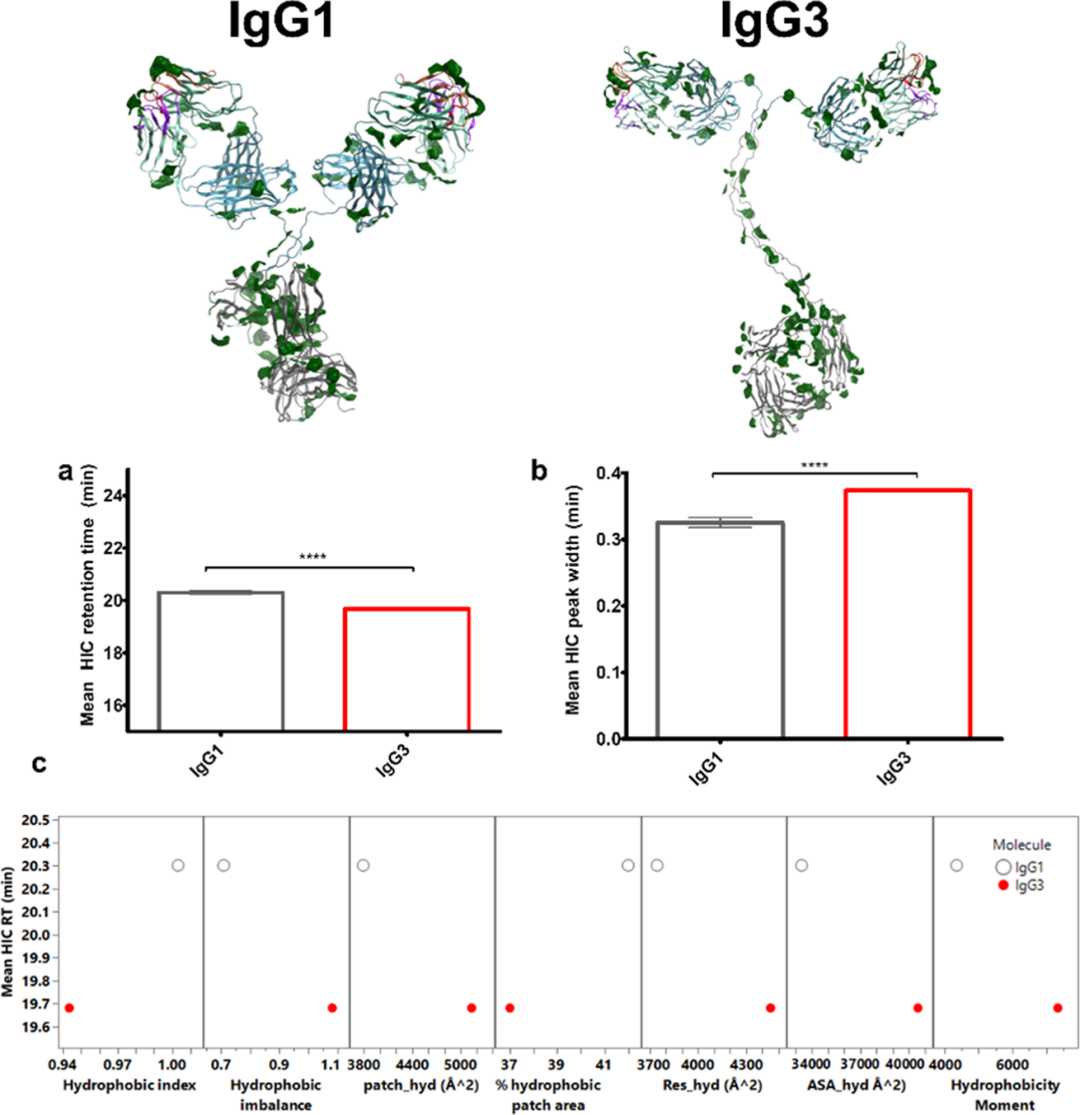
IgG3 exhibits a lower degree of hydrophobicity, contradicting computed solvent accessible hydrophobic area data. Protein patch surface maps for anti-IL-8 IgG1 and IgG3, filtered for hydrophobic patches (green). (a) Retention timeand and (b) peak width on the HIC columnbetween IgG1 and IgG3 are compared. (c) Pair-wise scatter plot comparisons between in silico descriptors and HIC retention time (RT). Unpaired *t* test **** denotes a *P* < 0.0001. Error bars represent standard deviation. Effect sizes were large (Cohen’s *d* > ± 0.8 standard deviations) for mean HIC RT (*d* = −13.1), and mean HIC peak width (*d* = 8.8).

To evaluate the hydrophobicity of anti-IL-8 IgG1 and IgG3, hydrophobic interaction chromatography (HIC) was used (Figure 6). A significantly lower on-column retention time (RT) was observed for IgG3 in comparison to IgG1 (Figure 6a), diagreeing with most hydrophobic-based in silico descriptors which showed higher predicted hydrophobicity for IgG3 in comparison to IgG1 (with the exception of a slightly lower hydrophobic index and proportional percentage hydrophobic patch area) (Figure 6c). IgG3 also presented increased peak broadening on the HIC column (Figure 6b), suggesting a potential increased population of different hydrophobic conformations.

#### Comparison of Anti-IL-8 IgG1 and IgG3 Colloidal Parameters

**Figure 7:**
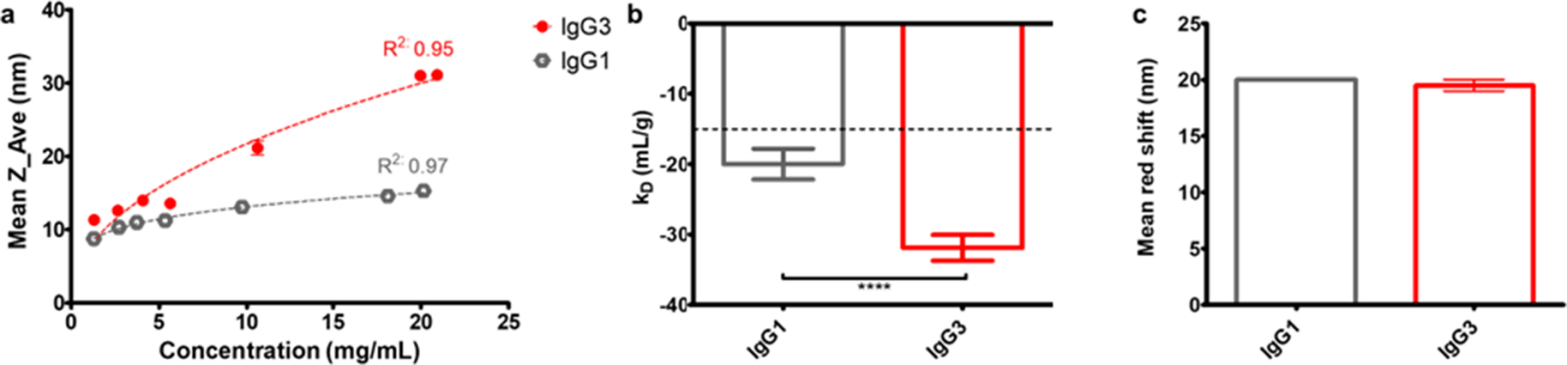
Colloidal interaction data from DLS measurements and AC-SINS for anti-IL-8 IgG1 and IgG3. (a) concentration-dependent measured z-average hydrodynamic diameter. Logarithmic fits of 10^(0.2log(concentration)+0.92)^ and 10^(0.46 log(concentration)+0.87)^ were applied to IgG1 and IgG3, respectively. Goodness of fit *R*^2^ values are reported. (b) Self-interaction parameter (*k*_D_) for IgG3. A dotted line at −15 mL/g represents the threshold for *k*_D_. (c) Mean red shift in absorbance spectra from AC-SINS (*N* = 2). Unpaired *t* tests were performed to determine significant differences between means (**** denotes a *P* < 0.0001). Error bars represent standard deviation. *N* = 3. Effect size was large (Cohen’s *d* > ± 0.8 standard deviations) for *k*_D_ (*d* = −1.5).

The concentration-dependent diffusion coefficient profile was measured for anti-IL-8 IgG1 and IgG3. We also used affinity-chromatography self-interaction nanospectroscopy (AC-SINS) (Figure 7) as an orthogonal approach to measure the comparative self-association behavior of IgG1 and IgG3. As expected, IgG3 measurements showed a larger hydrodynamic diameter (*Z*_ave) in comparison to IgG1, with a steady-concentration-dependent increase over the 1–20 mg/mL test concentration range (Figure 7a), which corresponded to slower diffusion coefficients (Supporting Information). The measured self-interaction parameter, *k*_D_, for both molecules, was negative and below the −15 mL/g threshold set, suggesting predominant attractive forces. However, the derived *k*_D_ parameter was significantly more negative for IgG3 anti-IL-8 compared with IgG1, indicative of increased self-association propensity. Conversely, IgG3 showed a red shift comparable to that in the AC-SINS assay, which did not correlate with the suggested increased self-association propensity from the DLS-derived k_D_ parameter.

#### Viscosity Predictions and Analysis

**Figure 8:**
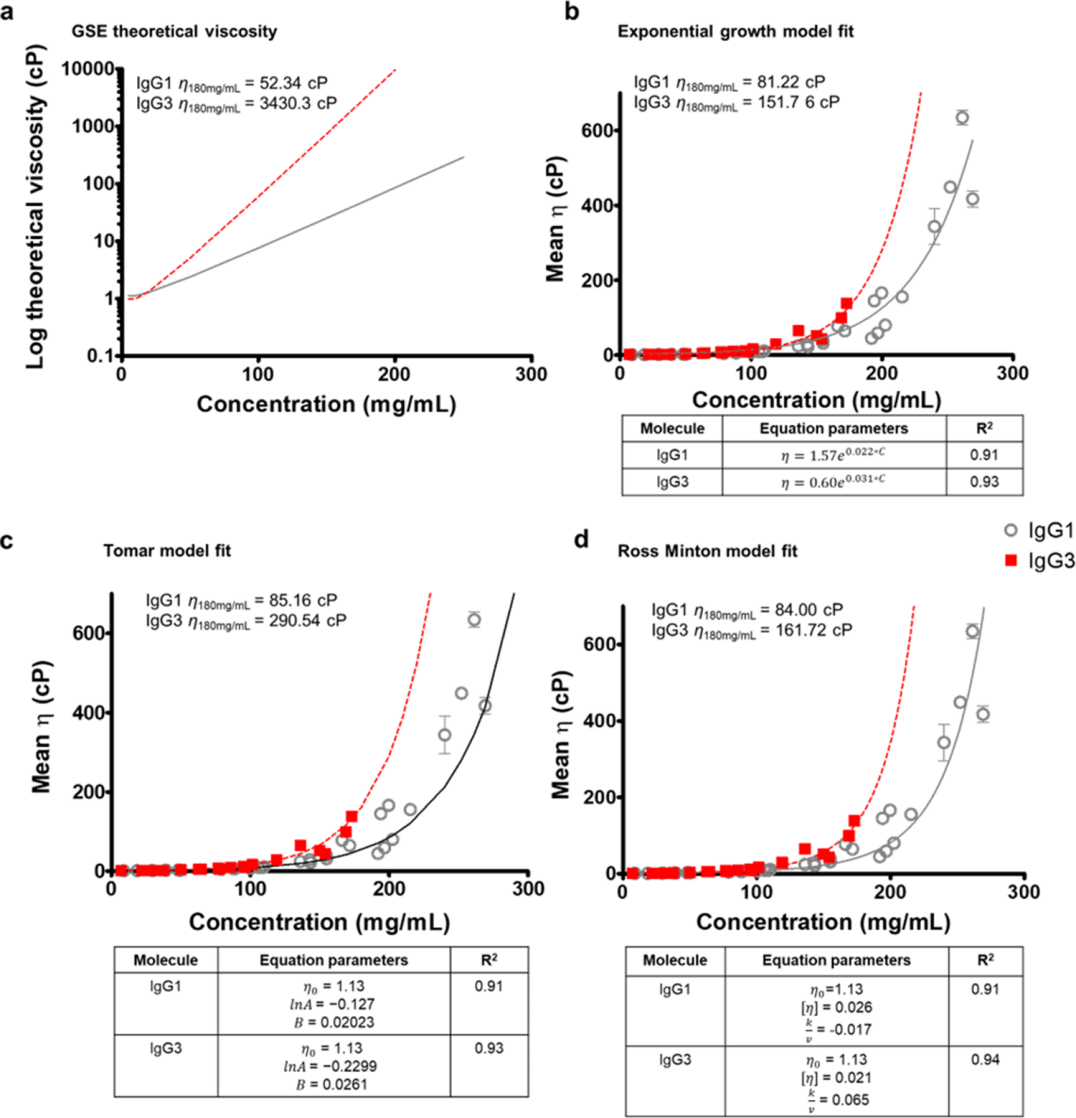
IgG3 has a higher apparent viscosity than IgG1 at high concentrations. (a) Generalized Stokes–Einstein equation was calculated from exponential extrapolation of diffusion coefficients and logarithmic fit of *z*-average diameters measured in the dilute range (1–20 mg/mL). Three viscosity model equations (lines) were used to fit the mean apparent viscosity data for IgG1 (gray circles) and IgG3 (black squares). (b) Exponential growth model (c), modified Ross Minton model, and (d) Tomar fit model. For each model, the predicted viscosity of 180 mg/mL is reported for both IgG1 and IgG3. Error bars represent standard deviation.

The generalized Stokes–Einstein viscosity (eq 4) was calculated using DLS-derived diffusion coefficients (Supporting Information) and hydrodynamic diameters (Figure 7a). The resulting theoretical viscosities (Figure 8a) were log-transformed and showed a distinct increased viscosity for IgG3 at formulation concentrations ≥50 mg/mL in comparison to IgG1. Overestimation of the IgG3 viscosity and underestimation of IgG1 viscosity at 180 mg/mL (3430 and 52 cP, respectively) is reflective of the derivation of data measured in the 1–20 mg/mL concentration regime and the assumptions of using exponential fits for the diffusion coefficients and logarithmic fits for the *Z*-average values.

Therefore, we also measured the apparent viscosities of IgG1 and IgG3 at concentrations up to 150 mg/mL (Figure 8b–d). We observed an elevated apparent viscosity for IgG3 compared to IgG1, in agreement with predicted theoretical viscosity and colloidal measurements. To compare the predictive power of different viscosity models, we fit the viscosity–concentration curves to three different models including an exponential growth model (eq 5), a Tomar model (eq 6), and a modified Ross–Minton model (eq 7). The exponential growth fit (Figure 8b) had a similar inflection point and gradient to the Ross–Minton fit (Figure 8d), resulting in similar viscosity interpolations at 180 mg/mL of 81.22 and 84 cP for IgG1, and 151.76 and 161.72 cP for IgG3, respectively. The Tomar model fit (Figure 8c) exhibited a shifted knee of the exponential curveand a steeper gradient compared with the two previous models, resulting in higher interpolated viscosity predictions at 180 mg/mL (85.16 cP for IgG1 and 290.54 cP for IgG3).

Finally, we examined the individual contributions from each molecule to the solution viscosity by calculating intrinsic viscosity, [η], from measurements in the low concentration regime (0–50 mg/mL; Table 2). Although statistically comparable to IgG1, IgG3 had an increased intrinsic viscosity, correlating with its increased hydrodynamic size. This suggests that the increased size and effective volume fraction of IgG3 increases the solution’s resistance to flow in the dilute regime.

**Table 2:** Intrinsic Viscosity and Huggins’ Coefficient (*k*_H_) for Anti-IL-8 IgG1 and IgG3^a^

molecule	intrinsic viscosity (mL/g)	*k*_H_
IgG1	8.28 (±3.89)	5.30 (±1.77)
IgG3	10.42 (±2.89)	1.27(±0.51)

a Mean ± standard errors are shown. *N* = 2.

Moreover, the Huggins’ coefficient (*k*_H_) was computed, describing the changes in the rate of viscosity increase from pairwise interactions. This has been previously equated to “solvent quality” with values >0.5 suggestive of “poorer solvents” that have solution viscosities more sensitive to protein–protein interactions (PPIs).^23^ Interestingly, IgG3 showed a reduction in *k*_H_ compared to IgG1, but both molecules had *k*_H_ > 0.5, indicating poor solvation.^23^

## Discussion

The choice of subclass during therapeutic mAb development plays a critical role in the desired efficacy, safety, and manufacturability of the drug product. Currently, IgG1 trends as the preferred subclass accounting for around 60% of all antibodies that are approved or in review.^26,27^ While IgG1 possesses enhanced physicochemical stability, solubility, reduced aggregation propensity, reduced afucosylation,^6,28^ and potency from high FcyR affinity,^29^ developability challenges are reported, particularly hinge-region fragmentation.^6^ Until now, the developability of IgG3 has been unexplored due to stability concerns, discounting its therapeutic potential with superior complement activation, high FCyR affinity, and hinge flexibility enabling engagement with previously inaccessible targets.^2,3^

In this work, we provide the first insights into the biophysical behavior of a recombinant anti-IL-8 IgG3, correlating *in silico* predicted molecular descriptors with experimental biophysical parameters and comparing these to a matched IgG1 with the same variable region sequence. Our goal was primarily to assess differences in physical stability and solution-phase viscosity-concentration profiles between these anti-IL-8 paired subclasses, while also predicting and measuring charge, hydrophobic, and colloidal parameters as known drivers of mAb developability issues. Using a combined computational and experimental approach, we have constructed a set of guidelines that could be used more widely for mAb developability.

### Reduced Physical and Conformational Stability of Anti-IL-8 IgG3

We compared the short-term physical and thermal stabilities of IgG1 and IgG3 (Figures 2 and 3), demonstrating a more rapid extent of monomer loss within a 57-day observation period. While there is a lack of published thermal stability data on IgG3, the IgG1 unfolding temperatures are broadly similar to published values for IgG1 molecules in prior developability studies.^30^ The extended hinge region of IgG3 is proposed to confer reduced *in vivo* stability, increased number of allotypes, and reduced half-life.^2 ,31 −33^ We hypothesize that the reduced domain unfolding temperatures we observed for IgG3, pair with the reported reduced conformational stability from the hinge region. Therefore, we propose additional structural analysis of anti-IL-8 IgG3 conformational stability to better understand its role in formulation shelf life prediction and reconcile these findings with functional stability and immunogenicity assessment. The immunogenicity of IgG3 resulting from concerns on glycosylation propensity has previously been flagged for this subclass,^2^ necessitating the monitoring of IgG3 post-translational modifications over time between for both batch-to-batch and shelf life stability.

### Predicted Charge Differences Do Not Translate to Differences in Isoelectric Points

Electrostatic surface potential mapping from homology constructs predicted an increase in the surface coverage of solvent-accessible negatively charged patches for anti-IL-8 IgG3 in comparison to IgG1, suggesting an increased likelihood for electrostatic interactions to occur (Figures 4 and 5). The theoretical isoelectric points (pIs) for IgG3 were predicted to be lower than those for IgG1. However, although slightly lower, the experimental pI for IgG3 was statistically comparable to that for IgG1. pI_3D showed greater predictive power than pI_seq for the anti-IL-8 full IgG models. Thorsteinson *et al.* similarly observed pI_3D to have the highest correlations to experimental parameters, but this was based on Fv models only and was statistically comparable to the sequence-based pI method.^34^ The increased negative patch count and area for IgG3 correlated with a decreased predicted net charge, which has been correlated previously with increased solution viscosity at dose-relevant formulation concentrations.^22,23,35^ Anti-IL-8 IgG3 showed a positive measured zeta potential (ζ) at pH 6.0 compared with a surprisingly negative potential for IgG1, which did not align with the *in silico* predictions of zeta potential and isoelectric points. The negative ζ for IgG1 may be accounted for by the preferential binding of anions to protein surfaces affecting the pH at which there is zero electrophoretic mobility, which has been reported as at least one pH unit below the pI determined from cIEF.^36^ The possibility of different conformational forms of IgG3 from inferred hinge flexibility with different ionic surface patch exposures may contribute to why a positive ζ was observed for IgG3. Furthermore, ζ is calculated from the electrophoretic mobility of the protein with an assumed spherical shape.^37^ It is also dependent on the orientation of the molecule in solution affecting the frequency shift of scattered light which may result in large discrepancies to the expected charge.^38^ Finally, *in silico* ζ predictions do not account for buffer composition affecting surface-bound ions or the effect of multiple or alternative species in solution (ζ measured at 5 mg/mL).

### Net Hydrophobicity of IgG3 Does Not Correlate with Predicted Hydrophobic Potential

Contrary to the predicted increased hydrophobic contributions from the hinge region both on a sequence level (with more cysteine, alanine, and proline residues) and on a structure level (with an increased hydrophobic area) (Supporting Information) anti-IL-8 IgG3 showed a shorter retention time on the hydrophobic-interaction chromatography column compared with IgG1 (Figure 6). We hypothesize that the discrepancies between predicted and experimental hydrophobicity may arise from changes in conformational forms of IgG3, varying exposure of the hinge residues and hydrophobic patches on the Fc. Increased net hydrophobicity has previously been correlated with increased solution viscosity occurring *via* cation-π and π–π stacking interactions from aromatic groups of solvent-exposed nonpolar amino acid residues.^17,39^ Moreover, increased hydrophobicity in the constant domain (Fc) of antibodies is widely correlated with a higher aggregation propensity, promoting an elevated mAb solution phase viscosity.^40,41^ In this case, as anti-IL-8 IgG3 showed a decrease in net hydrophobicity, we cannot attribute the increased self-association or aggregation propensity to hydrophobic interactions. Currently, there is a significant knowledge gap on the drivers of IgG3 hydrophobicity, both measured and predicted and how this affects the balance of domain–domain stability to unfolding propensity and aggregation.

### Increased Self-Association Propensity of Anti-IL-8 IgG3 Correlates with Hydrodynamic Size and Increased Viscosity

The self-interaction parameter, *k*_D_, is widely used for predicting the propensity for protein–protein interactions at the molecular level, which drives elevated solution viscosity at high mAb formulation concentrations. For both molecules, the *k*_D_ was negative and below the −15 mL/g arbitrary threshold set, suggesting predominant attractive forces. A more negative *k*_D_ was observed for anti-IL-8 IgG3 (Figure 7), indicating more attractive interactions between molecules in the dilute concentration regime compared with IgG1.^21,42−44^

Unexpectedly, the AC-SINS red shift for IgG3, another metric used to experimentally predict the mAb self-interaction propensity, showed an absorbance intensity profile comparable to that of the anti-IL-8 IgG1. We hypothesize that increases in red shift may be masked by the reduced binding of IgG3 to the anti-Fc conjugated gold nanoparticles used during AC-SINS measurements. This may be a result of conformational flexibility provided by the extended IgG3 hinge region, leading to structural blocking of adjacent binding sites on the nanoparticles. Subsequently, this could reduce the number of bound antibodies engaged in self-interactions.

Across all viscosity fitting models, an increased apparent viscosity was observed for IgG3 in comparison to IgG1, aligning with the decreased predicted net charge, increased negative patch distributions, and increased hydrodynamic self-associations (Figure 8). The extrapolation of the generalized Stokes–Einstein (GSE) model (Figure 8a) shows elevated viscosity, suggesting viscosity-contributing interactions in the dilute regime for anti-IL-8 IgG3. This aligns with the increased intrinsic viscosity for IgG3 (Table 2), suggesting that the increased hydrodynamic radius increases the fluid’s resistance to flow. Notably, no increase in Huggins’ coefficient (*k*_H_) was observed for IgG3, which suggests comparable protein–protein pairwise interactions that contribute to IgG1 viscosity. However, it is worthwhile to note the inaccuracies of the *k*_H_ parameter. The error in [η], from which the *k*_H_ parameter is derived, can arise from the use of simple linear regression of η_red_/*c* fits (Supporting Information) as well as interexperimental variability in viscosity measurements. Alternate nonlinear fits may be able to account for antibody molecules, which exceed the hard-sphere limit with regard to an effective volume fraction of >2.5. Another limitation of the Huggins’ coefficient is that it does not account for solvation effects in dilute antibody solutions.^23−25^

It is important to note that our homology constructs are based on one possible conformation, and particularly with the assumed structure of IgG3, there are risks of under- or overestimating the solvent-exposed surface potential. Our work uses these models as guiding tools to better understand mechanistic interactions that lead to molecular biophysical behavior. There are growing efforts to research different structural modeling tools as well as the use of molecular dynamics simulations with coarse grain simulation modeling^45,46^ that could help expand our knowledge of how both sequence and structure dictate interactions that lead to elevated viscosity and stability for IgG3.

## Conclusions

Preclinical developability assessment constitutes a prominent area of research for improving the probability of success for early-phase antibody candidates to reach clinical phases. Predictive tools probing the physicochemical and colloidal stability, affinity, and viscosity of antibodies in their formulation are being developed in combination with experimental assay pipelines, as well as machine-learning algorithms. This work defines a multiparameter set of guidelines for mAb using the context of the biophysical behavior of an anti-IL-8 IgG scaffold as exemplar. We provide the first insights into the biophysical behavior of a recombinant anti-IL-8 IgG3, comparing its computationally predicted molecular descriptors and experimentally determined parameters to that of a paired IgG1 with the same variable region sequence. Our goal was primarily to assess the differences in physical stability and solution-phase viscosity–concentration profiles for these anti-IL-8 paired isotypes as well as charge, hydrophobic, and colloidal parameters. It is recognized that the elevated solution viscosity of mAbs is driven by their self-association propensity. Hence, we used a combined *in silico* and comprehensive experimental pipeline to profile any viscosity differences between anti-IL-8 IgG1 and IgG3 molecules. We reconciled the predicted computational descriptors derived from the in silico homology model, including the sequence- and structure-based molecular descriptors determined for each anti-IL-8 molecule, with their measured biophysical properties.

Here, we find that the constant domain of anti-IL-8 IgG3 significantly influences its biophysical profile. IgG3 showed increased charge heterogeneity and self-association propensity, correlating with predicted increased and ionic surface potential from in silico homology modeling. This, alongside decreased physical and conformational stability, aligns with the elevated solution viscosity observed for IgG3 compared with IgG1. The increased hydrodynamic size of IgG3 correlated with increased intrinsic viscosity, supporting increased thermodynamic as well as hydrodynamic contributions to solution viscosity.

Our work uniquely defines the bounds of manufacturability in the context of the biophysical behavior of an IgG3 molecule. We demonstrate the reduced overall developability of an IgG3 to an IgG1 ortholog and recommend formulation optimizations and/or in-silico-directed sequence engineering to investigate the mitigation of such developability risks. We propose future investigations to use functional assays to support the use of the IgG3 subclass as a promising therapeutic modality.

## Supporting Information Available

Homology modeling of IgG3 (Tables S1 and S2); patch analysis and physicochemical descriptor definitions (Table S3); patch analysis of anti-IL-8 1 IgG1 and IgG3 (Tables S4 and S5); physicochemical descriptors for anti-IL-8 IgG1 and IgG3 (Table S6); analysis of identity by mass spectrometry (Table S7); scattering intensity profiles from nanodifferential scanning fluorimetry (nano-DSF) of anti-IL-8 IgG1 and IgG3 (Figure S1); diffusion coefficients from dynamic light scattering (DLS) for anti-IL-8 IgG1 and IgG3 (Figure S2); and intrinsic viscosity and the Huggins coefficient for anti-IL-8 IgG1 and IgG3 (Figure S3) (PDF)
